# INPATIENT AND OUTPATIENT PALLIATIVE CARE AMONG VETERANS: THE NATIONAL LANDSCAPE

**DOI:** 10.1093/geroni/igae098.0752

**Published:** 2024-12-31

**Authors:** Brystana Kaufman, Sandra Woolson, Madison Burns, Jessica Ma, Joshua M Thorpe, Susan Hastings, David Bekelman, Courtney Van Houtven

**Affiliations:** Duke University School of Medicine, Durham, North Carolina, United States; Durham VA Health Care System, Durham, North Carolina, United States; Durham VA, Durham, North Carolina, United States; Duke University School of Medicine, Durham, North Carolina, United States; VA Pittsburgh Healthcare System, Pittsburgh, Pennsylvania, United States; Durham VA, Durham, North Carolina, United States; Eastern Colorado Health Care System VA, Aurora, Colorado, United States; Duke University School of Medicine, Durham, North Carolina, United States

## Abstract

Palliative care (PC) improves quality of life for people with life-limiting conditions (LLC), which is common among older adults. In the United States, use of PC in the outpatient setting has expanded over the past decade; yet, most research has focused on inpatient PC. This study compared characteristics and use for veterans with inpatient or outpatient PC care nationally. The sample included veterans with specialty VA PC encounters, identified using VA and Medicare administrative data (2014-2017). Specialty PC encounters were identified using clinic stop codes (353, 351) and current procedural terminology codes (99242-99245). Of 120,249 unique veterans with specialty PC, 67.8% had PC only in the inpatient setting (n=81,523) and 32.2% had at least one PC encounter in the outpatient setting (n=38,726). One third experienced the first PC encounter within 2 years of meeting LLC criteria. Outpatient versus inpatient PC users were more likely to have cancer and less likely to have high frailty, but sociodemographic factors including rurality and housing instability were similar. Duration of hospice use was similar between inpatient (median = 37 days; interquartile range, IQR =11, 112) and outpatient (median=44 days; IQR=14, 118) PC users, and shorter among those with only one PC encounter (median =18 days; IQR=5, 64). Among veterans with PC use, one third received at least some PC in the outpatient care setting. Differences between veterans with inpatient and outpatient use motivates the need for further research to understand how care setting and timing of PC impacts outcomes for veterans and older adults.

